# Clinical efficacy and safety of full-dose versus half-dose corticosteroids plus leflunomide for IgA nephropathy

**DOI:** 10.1186/s12882-021-02555-z

**Published:** 2021-11-04

**Authors:** Yebei Li, Yi Xiong, Tianlun Huang, Xin Liu, Gaosi Xu

**Affiliations:** Department of Nephrology, The Second Affiliated Hospital of Nanchang University, No. 1, Minde Road, Donghu District, Nanchang, 330006 P.R. China

**Keywords:** IgA nephropathy, Leflunomide, Corticosteroids, Renin-angiotensin system blockers

## Abstract

**Background:**

The results of leflunomide (LEF) in patients with IgA nephropathy (IgAN) were inconsistent.

**Methods:**

A total of 149 kidney biopsy-confirmed IgAN patients with an estimated glomerular filtration rate (eGFR) ≥ 50 ml/min/1.73 m^2^ and protein excretion levels ≥0.75 g/d were enrolled, with 65 subjects receiving half-dose CS plus LEF (LEF group), and the 84 counterpart patients accepting full-dose corticosteroid (Full CS group). The primary outcomes included the complete remission (CR) rates and incidence of adverse events (AEs). The secondary outcomes were the overall remission (OR) rates and a combined event (eGFR reduced ≥30%, end-stage renal disease [ESRD], hemodialysis, peritoneal dialysis or kidney transplantation).

**Results:**

During the 18 months of follow-up, the CR rates were 72 and 64% in the LEF and Full CS groups (*P* = 0.299), respectively. The proportion of patients with OR rates in the LEF group and Full CS group was 89% versus 75%, respectively (*P* = 0.027). Serious AEs were observed only in the Full CS group (*P* = 0.017). The incidences of total AEs (*P* = 0.036) and infections (*P* = 0.024) were lower in the LEF group than in the Full CS group.

**Conclusions:**

LEF combined with half-dose CS is superior to full-dose CS in the treatment of IgAN.

## Introduction

Immunoglobulin A nephropathy (IgAN) is the most common primary glomerulonephritis worldwide [1]. With the progress of research, it has been found that the natural course of IgAN is far from benign, up to 30% of patients with IgAN will progress to kidney failure by 20 years [2]. The updated Kidney Disease Improving Global Outcomes (KDIGO) guidelines suggest that IgAN patients who remain at high risk of progressive chronic kidney disease (CKD) despite maximal supportive care are considered systemic glucocorticoids therapy for 6 months [3]. However, the long-term use of corticosteroids (CS) is associated with many serious adverse events (SAEs). And patients may not always respond to corticosteroid therapy leading to consideration of additive immunosuppressive therapies to obtain a synergistic effect [4].

A variety of immunosuppressants have been used for clinical treatment of IgAN, including cyclophosphamide (CTX), leflunomide (LEF), CS, azathioprine, mycophenolate mofetil, tacrolimus [5–7], etc. However, the protective role of immunosuppressive therapy was still in controversy [3, 8]. A multicenter RCT stated that the addition of immunosuppression to ongoing comprehensive supportive care in patients with high-risk IgAN did not significantly improve the outcome, and during the 3-year study period, more adverse reactions were observed among the patients who received immunosuppressive therapy [9]. LEF, an immunosuppressive medication that inhibits pyridine synthesis, has been widely used in rheumatoid and kidney diseases in recent years [10]. Several randomized trials demonstrated that LEF combined with low-dose CS is at least as effective as CS alone for the treatment of progressive IgA nephropathy, with fewer side effects [11, 12]. Besides, our previous research supported that half-dose CS plus renin-angiotensin system blockers (RASB) versus full-dose CS did not differ in terms of reducing proteinuria, but therapy with Half CS plus RASB resulted in fewer AEs in IgAN patients and might be a better option for IgAN [13].

Therefore, we conducted here a retrospective cohort study with long-term follow-up to evaluate the therapeutic effects and safety of half-dose CS plus LEF versus full-dose CS in patients with IgAN.

## Methods

### Ethical approval

This research was approved by the Regional Ethics Committee of Nanchang University Second Affiliated Hospital (No. [2020] 029) and was conducted according to the ethical principles contained within the Declaration of Helsinki. Due to the retrospective nature of the study, informed consent was abandoned. The design of the study fully considered the principles of security and fairness.

### Patients

For this retrospective, cohort study all cases kidney biopsy-confirmed IgAN from June 2011 to March 2020 at the Department of Nephrology, Nanchang University Second Affiliated Hospital, Jiangxi Province, China, were reviewed and included when meeting inclusion criteria. The following were required before entry into the study: (1) IgAN diagnosed by renal biopsy; (2) an age range of 16–65 years; (3) 24-h urinary total protein (24 h UTP) level > 0.75 g, (4) estimated glomerular filtration rate (eGFR) ≥ 50 ml/min per 1.73 m^2^, and (5) a follow-up time was up to 18 months. The exclusion criteria were: (1) rapidly progressive IgAN; (2) Henoch-Schönlein purpura nephritis, hepatitis-associated nephritis, lupus nephritis, or any other systemic disease known to be associated with secondary IgAN; (3) use of CS or other immunosuppressive therapy within the 6-month period before enrollment; (4) malignancy, hepatitis B and C virus and HIV infection, or acute central nervous system diseases, (5) abnormal glucose metabolism; (6) pregnancy, lactation, heart failure or severe infection.

### Treatment protocol

In China, the patient’s treatment plan (during hospitalization and follow-up) is conventionally based on the hospital’s routine clinical practice and the patient’s preferences. Some patients with IgAN are very worried about the adverse events (AEs) associated with full-dose CS, and hesitate to use this therapy and miss the best treatment time. Thus, we usually recommend the use of half-dose CS ± immunosuppressants therapy based on the hospital’s routine clinical practice. All enrolled patients had IgAN confirmed by renal biopsy. Patients in the LEF group (*n* = 65) who met the criteria received half-dose CS plus LEF, and the control group (Full CS group, *n* = 84) included all patients who received full-dose CS.

Before enrollment, all patients underwent a three-month run-in phase and adjusted strict supportive treatment (including RASB, low-salt diet and rigorous blood pressure control) according to proteinuria. Patients who had persistent proteinuria with urinary protein excretion levels ≥0.75 g/d were assigned to receive full-dose CS or half-dose CS plus LEF.

Patients in the LEF group received daily oral CS dosage starting from 0.4 to 0.6 mg/kg/day every morning for 2 months, and then decreasing by 20% each month for the next 4 months. LEF was orally administered with 50 mg/day for 3 days, reduced to 20 mg/day for 3–6 months, and subsequently tapered [5].

Patients in the CS group were treated with oral CS 0.8 to 1.0 mg/kg/day for 2 months and then tapered by 20% each month for the next 4 months. Subsequently, the steroid dose was slowly reduced again until it was withdrawn.

During treatment and follow-up, all patients received RASB unless or hypotension occurs (blood pressure was ≤90/60 mmHg). In addition, the use of other immunosuppressive therapies, such as azathioprine, cyclophosphamide or mycophenolate mofetil, was not allowed.

### Laboratory tests

Blood and urinary parameters were measured by the Department of Laboratory, Nanchang University Second Affiliated Hospital. At the beginning of treatment and at 3, 6, 12, and 18 months after treatment, we collected routine urine and blood biochemical data of patients, including 24 h UTP, liver function, standard complete blood count, serum creatine and eGFR.

### Follow-up and clinical evaluation

The treatment lasted for 12 months. At 6, 12, and 18 months after the initiation of the above-mentioned immunosuppressive therapy, we performed clinical efficacy evaluations and recorded the occurrences of any AEs.

The primary outcomes included the rates of complete remission (CR) and AEs. The secondary outcomes involved the rates of overall response (CR plus partial remission [PR]) (OR) and the incidence of a combined event (defined as eGFR reduced ≥30%, end-stage renal disease [ESRD], hemodialysis, peritoneal dialysis or kidney transplantation).

CR was defined as a 24 h UTP level < 0.4 g with a stable Scr level (defined as not more than 30% above baseline values). PR was defined as the achievement of 24 UTP greater than 0.4 g but less than 0.75 g and maintained at a stable Scr level.

### Statistical analysis

Statistical analyses were performed by Graph Pad Prism (version 7.0) and SPSS (version 23.0). Normally distributed variables were expressed as the mean ± standard deviation (SD) and were compared using an independent or paired *t*-test when appropriate. Nonparametric continuous variables were presented as the median of the interquartile range (IQR, 25th and 75th percentile) and nonparametric tests were used for comparison when appropriate. Categorical variables were summarized by proportions and employed by Pearson chi-square test. The Kaplan-Meier curve was used to describe the time-to-event data, and the difference between two groups was compared by the log-rank test. A *P* value of < 0.05 was considered statistically significant.

## Results

### Patient characteristics

**Fig. 1:**
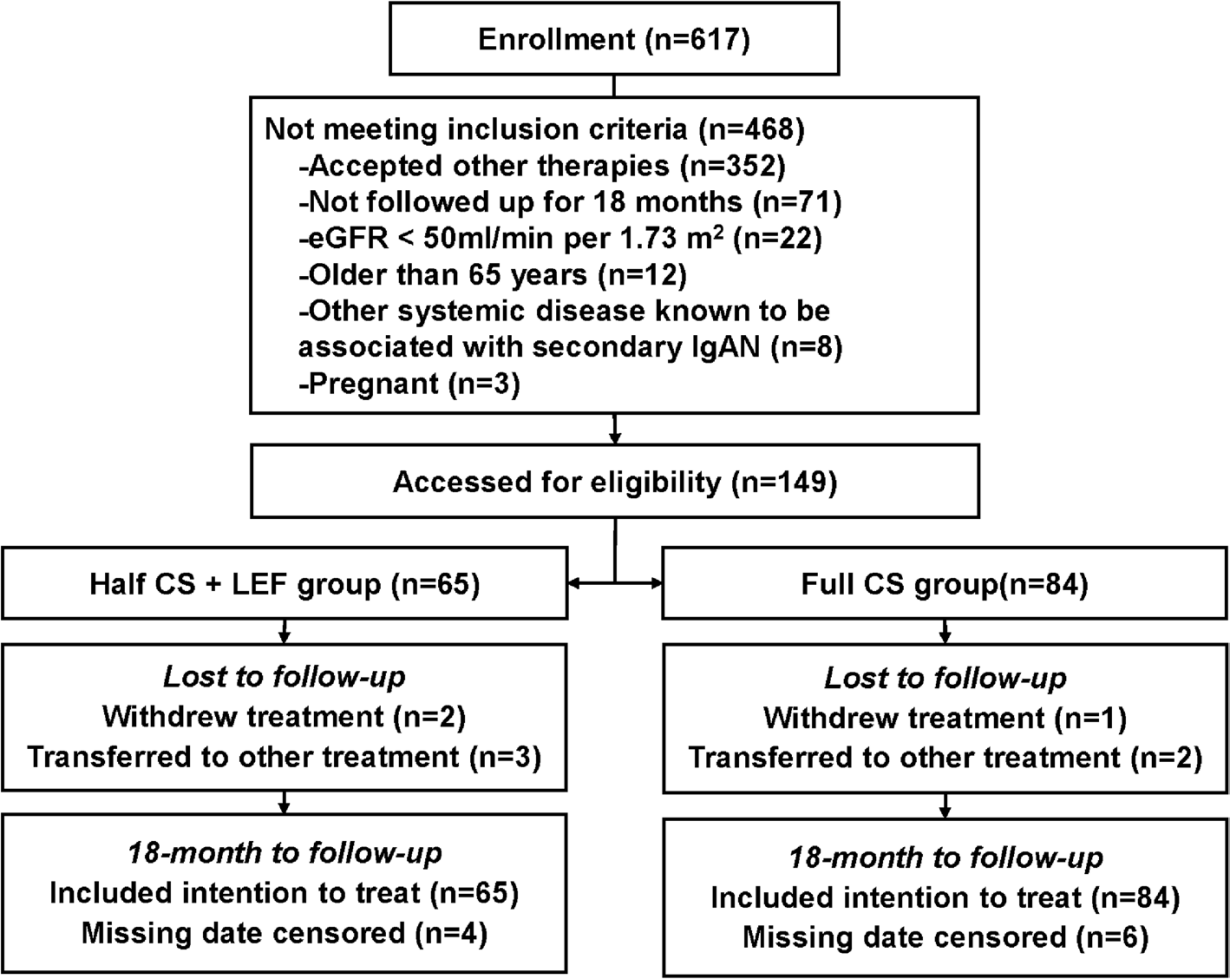
Flow diagram for inclusion of participants. Individuals who did not meet inclusion criteria were excluded. Of the remaining 167 patients, 65 with half-dose corticosteroid (CS) plus leflunomide (LEF) could be matched to 84 with full-dose CS only (3 patients withdrew treatments, 5 patients transferred to other treatment and 10 patients missed data censored during the observation period)

In total, 617 patients with type IgAN who had a 24 h UTP level > 0.75 g were identified (Fig. 1). We identified 149 patients that met the criteria (65 subjects received half-dose CS plus LEF, and the 84 counterpart participants with similar risk characteristics in terms of progression received full-dose CS). During the treatment and follow-up period, four subjects who received half-dose CS plus LEF lacked significant data (24 h UTP and Scr levels), while six patients in the Full CS group losted important data (Scr levels and medication records). Except for the above 10 people. Table 1 lists the baseline characteristics of two groups.

**Table 1:** Clinical features of participants at baseline

**Characteristic**	**Half CS + LEF (*n* = 65)**	**Full CS (*n* = 84)**	***P* Value**
Clinical characteristics at biopsy			
Men	28 (37)	45 (39)	0.204
Asian	65	84	–
Age (y)	32 ± 9.1	34 ± 9.5	0.205
Systolic blood pressure (mmHg)	117 ± 13.0	117 ± 10.7	0.556
Diastolic blood pressure (mmHg)	75 ± 9.3	76 ± 8.9	0.633
Serum creatinine (mmol/L)	93.01 ± 34.2	91.4 ± 42.8	0.404
eGFR (ml/min per 1.73 m^2^)	84.2 ± 30.5	86.7 ± 29.4	0.378
Serum albumin (g/L)	37.0 (35.0 to 39.8)	36.3 (33.9 to 38.6)	0.097
Urine protein (g/d)	3.03 (1.61 to 5.35)	3.10 (1.85 to 7.05)	0.151
Total cholesterol (mmol/L)	4.83 (4.23 to 5.39)	4.96 (4.21 to 5.68)	0.283
Triglycerides (mmol/L)	1.49 (1.06 to 2.12)	1.92 (1.00 to 2.80)	0.112
Antihypertensive			
RASB under follow-up	65	84	–
CCB under follow-up	8 (12)	12 (14)	0.725
β-receptor antagonists under follow-up	10 (15)	9 (11)	0.397
Pathologic			
Global glomerular sclerosis, %	2.7 (0.6 to 10.5)	3.0 (0.8 to 12.0)	0.214
M1	15 (23)	23 (27)	0.550
E1	18 (28)	19 (23)	0.477
S1	23 (35)	30 (36)	0.967
T1	6 (9)	10 (12)	0.646
C0	63 (97)	79 (94)	0.411
C1	2 (3)	5 (6)	0.411

Values for categorical variables were given as count (percentage); values for continuous variables, as mean ± standard deviation or median (IQR)

*Abbreviations*: *eGFR* Estimated glomerular filtration rate, *IQR* Interquartile range, *CS* Corticosteroid, *LEF* Leflunomide, *RASB* Renin-angiotensin system blockers, *CCB* Calcium channel blocker

### Effectiveness

**Fig. 2:**
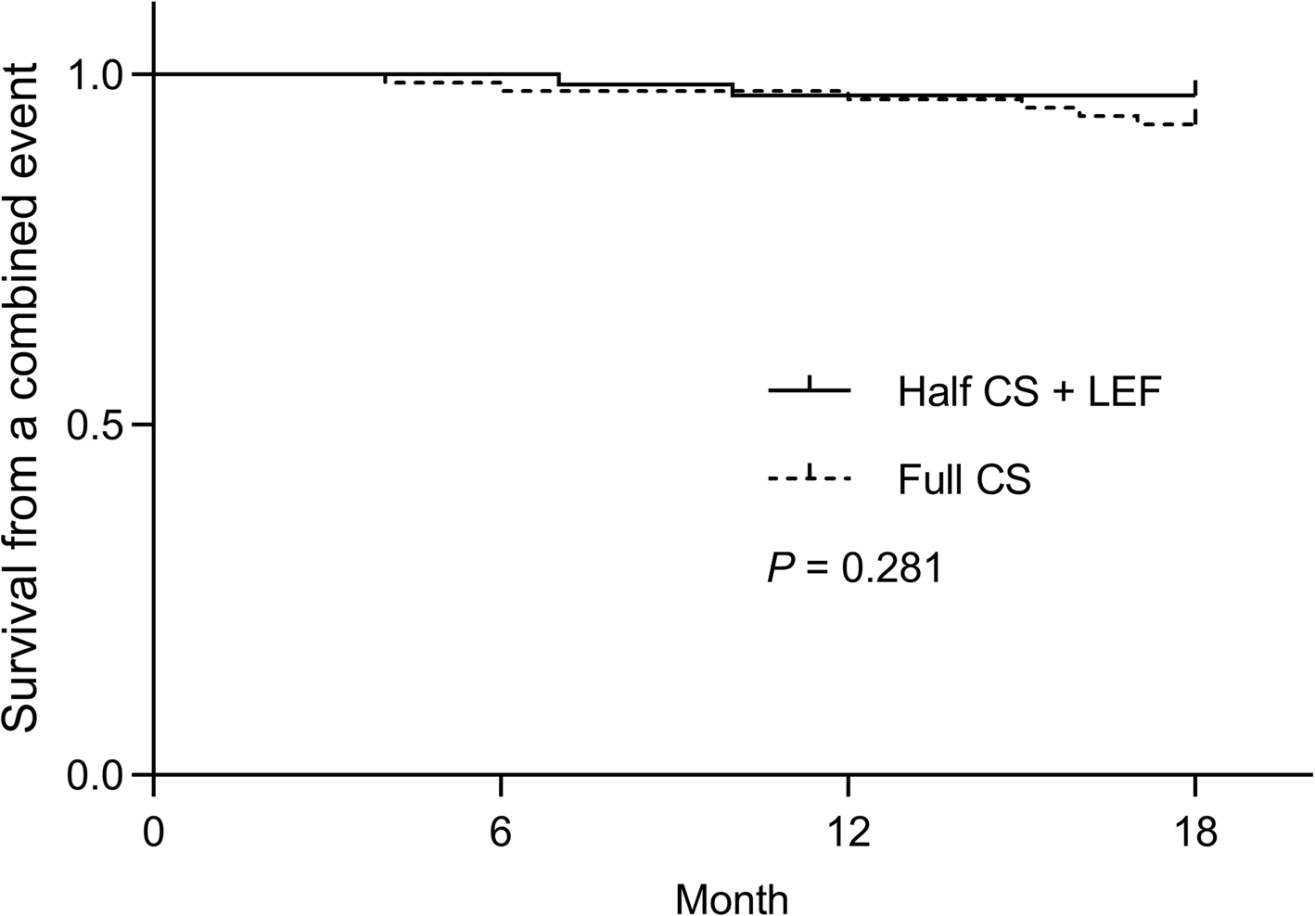
Cumulative incidence curves for the combined outcome in patients with IgA nephropathy treated with full-dose versus half-dose corticosteroid plus leflunomide

Over the 18 months of follow-up, the eGFR of two patients (3%) decreased more than 30% compared to the baseline in the LEF group, whereas six patients (7%) were observed in the Full CS group (*P* = 0.275, Table 2). Until the end of the study, neither group of patients had ESRD or required renal replacement therapy, and there was no significant difference between the two cohorts on the cumulative incidence curves of the combined outcomes (*P* = 0.281, Fig. 2).

**Table 2:** End points on the basis of the available patients at the end of the study phase

**End Point**	**Half CS + LEF (*n* = 65)** **End Point Value**	**Full CS (*n* = 84)** **End Point Value**	***P* Value**
*Combined event*			
eGFR decrease ≥30% (ml/min per 1.73 m^2^)	2 (3)	6 (7)	0.275
Onset of ESRD	0 (0)	0 (0)	–
Renal replacement therapy	0 (0)	0 (0)	–
*Primary outcomes*			
Complete remission at month 6	33 (51)	34 (40)	0.210
Complete remission at month 18	47 (72)	54 (64)	0.299
*Secondary outcomes*			
Overall remission at month 6	50 (77)	49 (58)	0.017
Overall remission at month 18	58 (89)	63 (75)	0.027

*Abbreviations*: *ESRD* End-stage renal disease, *CS* Corticosteroid, *LEF* Leflunomide

**Fig. 3:**
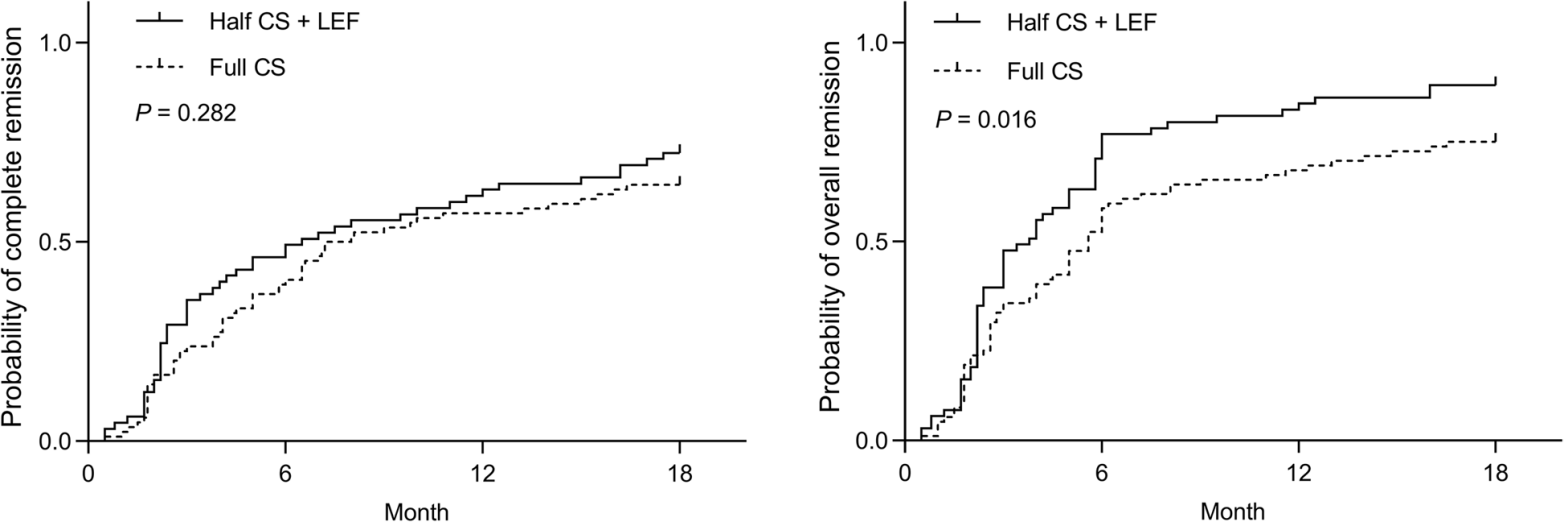
Kaplan-Meier analysis for the probability of complete remission (right) and overall remission (left) in patients with IgA nephropathy treated with full-dose versus half-dose corticosteroid plus leflunomide

At the 6th month, the proportion of CR in the LEF group and the Full CS group was 51% vs. 40%, respectively (*P* = 0.275). At the 18th month, the proportion of CR in the LEF group and control group was 72% vs. 64%, respectively (*P* = 0.299). The median time to CR in the LEF group was 6.5 months, and in the Full CS group was 7.6 months. At the end of the follow-up, there was no statistical difference in the Kaplan-Meier analysis of the probability of CR between the two cohorts (*P* = 0.282, Fig. 3).

The OR rates were 77% (50 of 65 patients) in the LEF group and 58% (49 of 84 patients) in the Full CS group at 6 months. There was a statistical difference between the two therapies (*P* = 0.017, Table 2). At the 18-month follow-up, the OR rates were 89% (58 of 65 patients) and 75% (63 of 84 patients) in the LEF and Full CS groups (*P* = 0.027), respectively. The median time to OR in the LEF group was 3.8 months, and in the Full CS group was 5.6 months. Between the two cohorts, the Kaplan-Meier analysis for the probability of OR was statistically difference (*P* = 0.016, Fig. 3).

### Adverse events

Table 3 lists the AEs that occurred during the treatment period. In the LEF group, 15 of 65 patients (23%) suffered from at least one first adverse event, whereas 33 of 84 patients (39%) were observed in the Full CS group (*P* = 0.036, Table 3).

**Table 3:** Summary of adverse events

**Primary outcomes**	**Half CS + LEF (*n* = 65)**	**Full CS (*n* = 84)**	***P* Value**^**a**^
Total SAEs	0 (0)	7 (8)	0.017
Pneumonia	0 (0)	5 (6)	0.045
Acute kidney injury	0 (0)	1 (1)	0.377
Osteonecrosis of the femoral head	0 (0)	1 (1)	0.377
ESRD	0 (0)	0 (0)	–
Total AEs^b^ (including SAEs)	15 (23)	33 (39)	0.036
Increase of liver enzymes (i.e., ALT> 50 IU/ml)	6 (9)	6 (7)	0.642
Leukopenia	3 (5)	1 (1)	0.200
Alopecia	2 (3)	0 (0)	0.106
Newly diagnosed diabetes	2 (3)	6 (7)	0.275
Gastrointestinal symptoms	1 (2)	4 (5)	0.279
Infections^c^	11 (17)	28 (33)	0.024
Pneumonia	0 (0)	5 (6)	0.045
Upper respiratory tract infection	7 (11)	14 (17)	0.305
Varicella zoster virus	1 (2)	1 (1)	0.855
Urinary tract infection	2 (3)	2 (2)	0.794
Other infections	1 (2)	6 (7)	0.109

Unless otherwise indicated, values were given as number (percentage). Includes all matched patients who received at least 1 dose of the study drugs. Terms used to describe AEs were those listed in the Common Terminology Criteria for Adverse Events, version 4.0. Multiple occurrences of the same AE in 1 person were only counted once

*Abbreviations*: *AE* Adverse event, *SAE* Serious adverse event, *ESRD* End-stage renal disease, *CS* Corticosteroid, *LEF* Leflunomide, *ALT* Alanine aminotransferase

^a^*P* value for comparisons between the number of patients in the Half CS + LEF group and the number of patients in the Full CS group

^b^Number of patients with at least one event

^c^The categories under “Infections” were not mutually exclusive

No patients died during the follow-up. In the Full CS group, seven SAEs were observed: five cases of pneumonia, one case of acute kidney injury, and another case of serious femoral head necrosis. No SAEs occurred in the LEF group. Among the AEs observed in the two cohorts, more than half of the events were related to infections. The results showed that compared with the Full CS group, the incidence of infection in the LEF group was significantly lower (17% [11 of 65] vs 33% [28 of 84], *P* = 0.024).

The proportion of patients with abnormal liver function in the LEF group and Full CS group was 9% versus 7%, respectively (*P* = 0.642). Between the two groups, the leukopenia in the LEF and Full CS groups was 5 and 1% (*P* = 0.200), the alopecia was 3 and 0% (*P* = 0.106), the newly diagnosed diabetes was 3 and 7% (*P* = 0.275), and the gastrointestinal symptom was 2 and 5% (*P* = 0.279).

## Discussion

IgAN is the main cause of primary glomerulonephritis, and its treatment options remain limited. Therefore, there is an urgent need to improve treatment methods to alleviate this condition. Although the etiology and pathogenesis of IgAN are unclear, IgA-dominant deposition in the mesangial area has been proposed as the critical factor in the onset of IgAN, which might promote the active and potentially reversible use of immunosuppressive treatment [9, 14, 15]. The updated KDIGO guidelines suggested that all IgAN with proteinuria > 0.5 g/24 h, irrespective of whether they have hypertension, are treated with either an ACEi or ARB [3]. However, the current evidence about the different immunosuppression therapies remains to be elucidated [3, 4, 7]. Thus, we first conducted a retrospective cohort study with 149 IgAN patients to evaluate the effectiveness and safety of half-dose CS plus LEF vs. full-dose CS.

LEF is an immunosuppressive agent inhibiting T- and B-cell functions which has long been used in rheumatology [10, 11]. Its mechanism of action involves inhibition of dihydroorotate dehydrogenase, as well as a number of tyrosine kinase signaling molecules involved with immune function [16]. Through the above-mentioned mechanism, LEF can inhibit serum IgG and IgM levels, thereby reducing the production of inflammatory mediators, inhibiting the proliferation of smooth muscle cells, and decreasing the production of free radicals in immune cells [10, 11, 16].

Previous investigations demonstrated that LEF could attenuate inflammation and improv kidney injury. *Lou* et al. explored the effect of LEF for treatment of IgAN [11]. *Min* et al. reported that LEF combined with low-dose corticosteroid was at least as effective as corticosteroid alone for the treatment of progressive IgAN, and had fewer SAEs [12]. Our present research indicates that, compared with the full-dose steroid monotherapy, half-dose CS plus LEF can effectively improve the overall response, reduce proteinuria in the treatment of IgA, and with fewer AEs. A recent meta-analysis of 44 studies involving 1802 patients compared LEF (plus steroid or ACEi) with steroid therapy alone [17]. Consistent with our findings, LEF showed a marked advantage in improving renal function and safety, as compared with steroid ACEi therapy alone.

The AEs of LEF include elevated of liver enzymes, leukopenia, alopecia and gastrointestinal syndrome. Compared with the SAEs associated with full-dose CS, LEF has relatively mild side effects, which indicates that LEF is relatively safe for the treatment of IgAN. In our study, the incidence of AEs was similar between the Full-CS and LEF groups. One point to emphasize is that although this is an infrequent adverse event, hepatotoxicity is the main concern with the use of LEF in clinical practice, being necessary a near analytic follow up after the therapy instauration (every 2 weeks).

We observed no statistical differences regarding combined outcome between the two cohorts. Consistent with our study, *Min* et al. found that LEF combined with low-dose corticosteroid, after 88 months of follow-up, was as effective as corticosteroids alone in renal survival [12]. Several previous studies have manifested that compared with conventional steroid monotherapy, immunosuppressive therapy alone or in combination with CS has the same or better long-term efficacy in treating IgAN [8, 17, 18].

There were several limitations to this study. Firstly, this is a single-center study with a relatively small sample size. Therefore, we could not perform subgroup analysis and true differences in kidney survival between the groups might thus be masked. Secondly, missing data were inevitable. However, they would tend to bias the results toward the null hypothesis. Thirdly, the course of therapy and follow-up were quite short, thus the long-term effect of LEF remains to be negotiated.

## Conclusion

In conclusion, during our study, LEF combined with half-dose CS seems to be at least as effective as full-dose CS for the treatment of IgAN, and showed a higher OR rate and fewer AEs. For IgAN patients who have full-dose CS relative contraindications or are concerned about their complications, this regimen may be a better choice.

## Data Availability

The datasets used and/or analysed during the current study are available from the corresponding author on reasonable request.
